# Bilateral Lumbar Facet Synovial Cysts as a Cause of Radiculopathy

**DOI:** 10.1155/2022/2519468

**Published:** 2022-11-07

**Authors:** Pawin Kasempipatchai, Verapan Kuansongtham, Monchai Ruangchainikom, Khin Myat Myat Lwin

**Affiliations:** ^1^Department of Spine, Bumrungrad International Hospital, Bangkok, Thailand; ^2^Department of Orthopaedic Surgery Faculty of Medicine, Siriraj Hospital, Mahidol University, Thailand

## Abstract

Remarkable advancements in endoscopic spinal surgery have led to successful outcomes comparable to those of conventional open surgery with the benefits of less traumatization and postoperative spinal instability. Bilateral lumbar facet cysts are rarely found in the spinal canal. We report a rare case of L4–L5 bilateral lumbar facet cysts compressing the nerve root in a patient who presented with L5 radiculopathy. Endoscopic decompression and removal of the cysts without fusion were performed. Histopathology revealed synovial cysts. Postoperatively, the patient showed a total resolution of symptoms with sustained benefits at the final evaluation. No recurrence of pain and no further segmental instability were observed at the 1-year follow-up.

## 1. Introduction

Synovial cysts most commonly involve the joints of the extremities. However, they are rarely found in the spinal canal and are uncommon causes of radicular pain [1]. Because of their rarity, synovial cysts are easily misdiagnosed in cases of persistent low back pain and radiculopathy [2]. This article describes a case of bilateral, symptomatic lumbar facet cysts treated with advanced, full-endoscopic surgical techniques. These techniques provide successful outcomes that are comparable to those of conventional open surgery [3, 4]. To the authors' knowledge, only 1 case of bilateral lumbar facet cysts was previously reported as having been caused by calcium pyrophosphate deposition [5].

## 2. Case Report

A 59-year-old male presented with right buttock pain radiating down the right posterior thigh to the calf and numbness down to the right big toe for a month. He stated that he felt pain with all body positions. His self-reported pain scores were 2 for the back and 6 for the right leg. Before coming to our spine clinic, he had consulted a doctor at another hospital.

Grade 1 spondylolisthesis at L4–L5 and a large synovial facet joint cyst at left L4–L5 were detected on magnetic resonance imaging (MRI). Consequently, the patient was administered a transforaminal epidural steroid injection into the right L4–L5. He said 70% of his pain had been relieved three days after the injection. Physical examination showed a negative straight-leg raising test at the angle of 90 degrees on both sides and no weakness of the lower extremities.

**Figure 1:**
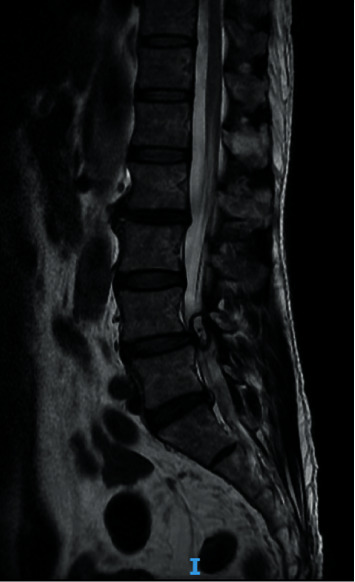
Preoperative magnetic resonance imaging (MRI) scan. This sagittal T2-weighted MRI image shows a hyperintense cystic lesion on the left side at the L4–5 level.

**Figure 2:**
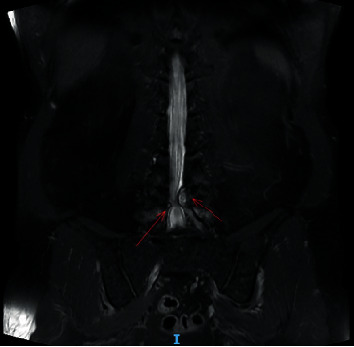
Preoperative magnetic resonance imaging (MRI) scan. This coronal T2-weighted MRI image shows cystic lesions on both sides, with each lesion arising from the right L4–5 facet joint.

**Figure 3:**
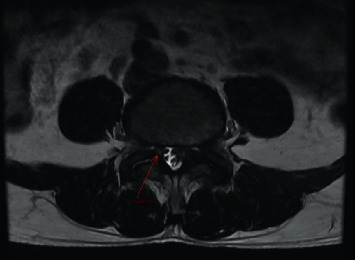
Preoperative magnetic resonance imaging (MRI) scan. This axial T2-weighted MRI image shows a cystic lesion (red arrow) arising from the right L4–5 facet joint, causing the narrowing of the right neural foramina. Bilateral facet synovial cysts are also evident.

Nevertheless, the patient's pain level did not decrease after 2 months of treatment with medication and physiotherapy. He was advised to have a repeat MRI scan and an X-ray of the lumbosacral spine. The new scan indicated that the size of the left facet cyst at L4–L5 was unchanged (1.1 cm). However, its wall was thicker, and its internal content was clearer than before (Figure 1). There was also left facet hypertrophy and a diffuse bulging disc. Together, these had caused mild central canal stenosis and severe left L5 lateral recess stenosis, with left L5 traversing nerve root compression (Figures 2 and 3).

**Figure 4:**
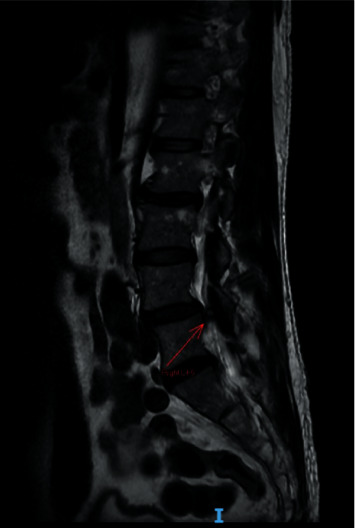
Preoperative magnetic resonance imaging (MRI) scan. This sagittal T2-weighted MRI image shows a hyperintense cystic lesion on the right side at the L4–5 level.

**Figure 5:**
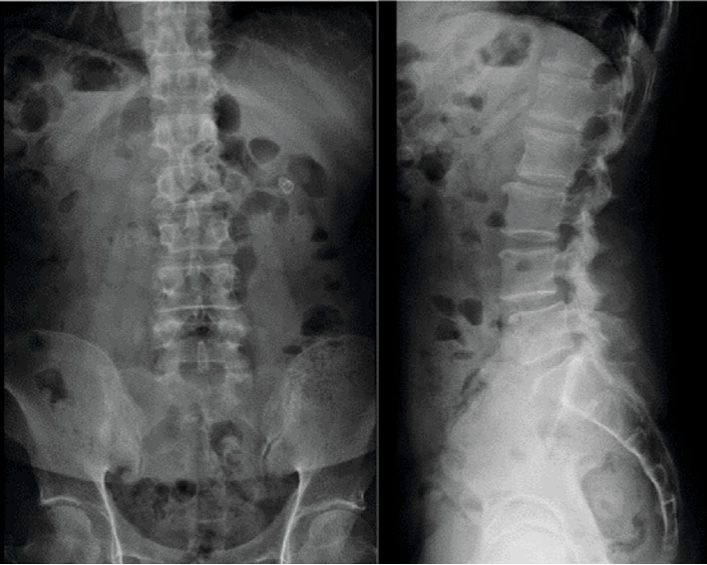
Preoperative X-ray film. These anteroposterior and lateral views reveal grade 1 spondylolisthesis of L4 over L5.

**Figure 6:**
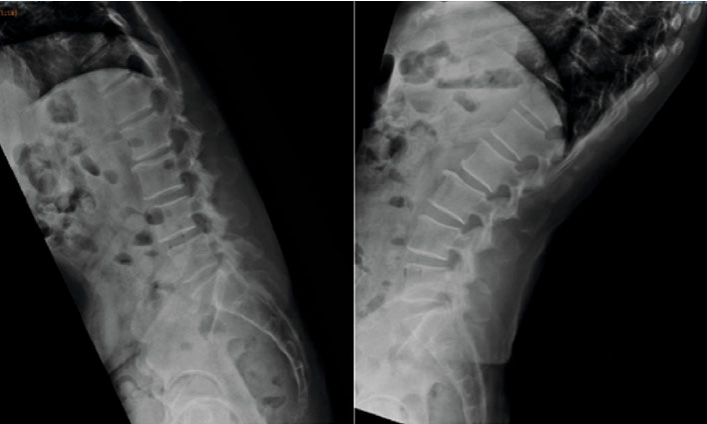
Preoperative X-ray films. No further instability of L4 over L5 is evident in upright lateral flexion and extension views.

A persistent, smaller, right facet synovial cyst was also noted. Its size was unchanged at approximately 0.5 cm (Figure 4), causing right lateral recess stenosis with right L5 traversing nerve root compression. There was also a grade 1 spondylolisthesis of L4 over L5 (Figure 5). Upright lateral flexion and extension X-rays of lumbosacral spine showed no further instability of L4 over L5 (Figure 6).

**Figure 7:**
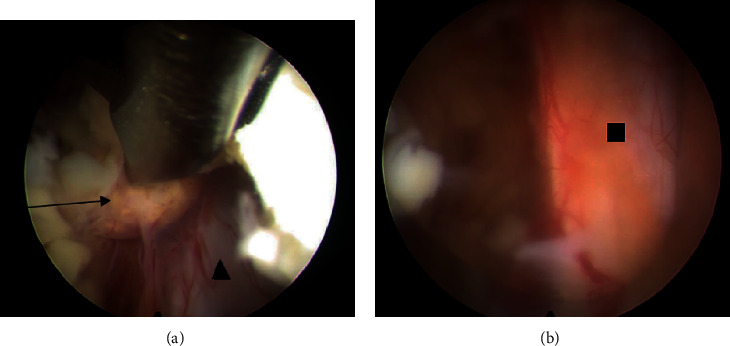
(a) Intraoperative image. It shows the removal of the facet cyst (black arrow) alongside the neural structure (▲). (b) Intraoperative image. No neural structure compression remains after cyst removal (∎).

Given the findings, we planned a full-endoscopic decompression of right L4–L5 with cross-decompression to remove the facet cysts. The patient underwent the procedure, with the bilateral cysts at L4–L5 removed via the interlaminar technique (right-side approach). Operative findings revealed a small facet cyst causing severe right L5 compression at the lateral recess. There was a slight adhesion between the nerve and the cyst. On the left L4–L5, a large facet cyst was compressed the thecal sac, but there was no compression of the left L5 nerve. There was more adhesion to the thecal sac on its left side than on its right side (Figures 7(a) and 7(b)). Pathology showed facet cysts, each showing mild, chronic inflammation of the walls.

**Figure 8:**
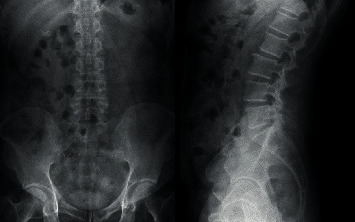
Postoperative X-ray films at the 1-year follow-up. Increased instability is not apparent in either the anteroposterior or the lateral view.

**Figure 9:**
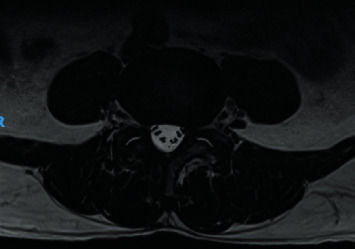
Postoperative magnetic resonance imaging (MRI) scan at the 1-year follow-up. This axial T2-weighted MRI image confirms the absence of bilateral cysts.

**Figure 10:**
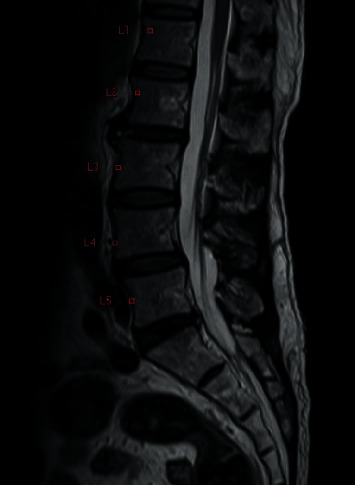
Postoperative magnetic resonance imaging (MRI) scan at the 1-year follow-up. This sagittal T2-weighted MRI image confirms the absence of bilateral cysts.

After surgery, there was neither surgical site pain nor right leg pain. Numbness was improved. The patient recovered well from the surgery and was discharged the next day. The wound was dry and clean without signs of infection. The follow-up for this case was 1 year. During that period, the patient remained free of pain in his back and right leg. Postoperative lumbosacral spine X-ray films and MRI at 1 year showed no further increase in segmental instability (Figure 8) and the absence of bilateral cysts (Figures 9 and 10).

## 3. Discussion

Synovial and ganglion cysts were first described as juxtafacet cysts by Kao et al. in 1974 [6]. Synovial cysts protrude into the synovial lining through a defect or rupture of a degenerated facet joint. Their pathogenesis is not well understood and remains a topic of debate. The cysts are common in older adults, with patients having an average age of 66 years [7]. In a retrospective review of MRIs from 303 patients, Doyle and Merrilees found a prevalence of 2.3% for anterior spinal cysts and 7.3% for posterior spinal cysts [8]. In the lumbar spine, synovial facet cysts are most commonly found at the L4–L5 level (68.4%). The L4–L5 level is generally regarded as the most mobile of all vertebrae levels and is frequently associated with spondylolisthesis [8, 9]. Bilateral lumbar facet cysts are rare.

The clinical presentation of facet cysts depends on their size, site, and relationship to adjacent structures [10–12]. Patients may present with back pain, radiculopathy, and claudication but rarely cauda equina syndrome. MRI is the modality of choice for diagnosing lumbar facet cysts [13, 14]: it has a 90% sensitivity compared with the 70% for computed tomography [15, 16]. Lumbar facet cysts generate a range of MRI signals depending on the often variable content of the cysts [17]. A typical synovial cyst appears with a low-intensity signal in T1-weighted images and a high-intensity signal in T2-weighted sequences [18]. In our patient, we performed a preoperative lumbar spine MRI (Figures 1–4).

Even though there have been reports of synovial cysts resolving spontaneously, they usually require treatment [19]. However, the optimal treatment remains unclear. Nonsurgical treatment can be considered the first option, but its results can be disappointing. In a large European study on 77 patients, the failure rate of conservative treatment reached 60% by 6 months, and patients were eventually treated with surgery [18]. A retrospective analysis of 30 nonsurgically treated patients revealed that only 33% (10) had excellent or good outcomes 6 months after treatment, whereas 47% (14) had to undergo surgery [20, 21]. Epidural injections of corticosteroids or injections into the facet joint may reduce the inflammatory process and resolve symptoms in up to 70% of patients. Unfortunately, the effectiveness of these approaches is only temporary [22].

Surgical treatment is recommended as soon as conservative treatments fail to control symptoms or if neurological deficits develop. The surgical techniques vary. Trummer et al., Lyons et al., and Hellinger and Lewandrowski reported that performing a medial facetectomy or hemilaminectomy alone was associated with an increased incidence of back pain and cyst recurrence, and the literature debates whether these techniques produce spinal instability [2, 7, 23]. Decompression with instrumented fusion appears to be associated with the lowest incidences of cyst recurrence and back pain [18, 24]. Controversy exists about fusion as a first-line treatment. Sabo et al. found no difference in outcomes for patients undergoing fusion compared with those treated with cyst excision alone [25]. Cyst excision alone as first-line treatment seemed sufficient for 88.6% of the treated patients. Thus, spondylolisthesis is not an absolute indication for arthrodesis when operating on synovial cysts [7].

In our reported case, we performed a full-endoscopic decompression and removed the bilateral cysts via the interlaminar technique without fusion. This approach was used because lumbosacral X-ray films showed only a grade 1 spondylolisthesis, and the upright lateral flexion-extension films showed no instability. The major advantage of a full-endoscopic decompression is that it completely removes bilateral cysts with minimum disruption to ligamentous and bony structures. The procedure thereby decreases the risk of progressive instability and the need for fusion. In addition, this technique requires an incision of less than 1 cm, resulting in early ambulation and a short hospital stay [23, 26, 27]. Although full-endoscopic decompression has several tangible benefits, the initial learning curve for this interlaminar procedure is steep [28]. To flatten the curve, Choi et al. [29] recommended that the first 10 cases be performed under the supervision of an experienced surgeon. Trainees should gain adequate experience by starting with simple cases in which no serious problems are anticipated from the anatomical conditions [30].

The present study shows the results of a full-endoscopic interlaminar operation. The reduced resection of spinal structures achieved with the full-endoscopic technique produces less operation-induced segment instability [31]. In an analysis of 48 patients treated with endoscopic removal of facet cysts, Hellinger and Lewandrowski reported that excellent and good Macnab outcomes were obtained by 77.1% of patients. The remaining patients (22.9%) had fair outcomes that were significantly associated with segmental instability of the involved lumbar facet joint (*P* < 0.001) [23]. With our reported case, sustained benefits were apparent 1 year postoperatively without recurrence of the cysts or further instability of the lumbar spine.

The authors declare that there are no potential conflicts of interest relating to this article's research, authorship, or publication.

## 4. Conclusions

Unilateral lumbar facet cysts are rare, and bilateral lumbar facet cysts are even more rarely found in the spinal canal. The authors suggest that an endoscopic interlaminar approach offers several advantages. It might result in less postoperative spinal instability than conventional open surgery, even when bilateral cysts are completely removed, with minimal soft tissue traumatization and bleeding, quick rehabilitation, and a short hospital stay. The authors believe this technique has the potential to provide suitable outcomes and is a safe option compared with both open and microscope-assisted surgery.

## Data Availability

The data used to support the findings of this study are included within the article.
